# Synergy Effect of Au and SiO_2_ Modification on SnO_2_ Sensor Properties in VOCs Detection in Humid Air

**DOI:** 10.3390/nano10040813

**Published:** 2020-04-23

**Authors:** Dayana Gulevich, Marina Rumyantseva, Evgeny Gerasimov, Nikolay Khmelevsky, Elena Tsvetkova, Alexander Gaskov

**Affiliations:** 1 Chemistry Department, Moscow State University, 119991 Moscow, Russia; dayana-nsu@mail.ru (D.G.); gaskov@inorg.chem.msu.ru (A.G.); 2 Boreskov Institute of Catalysis SB RAS, 630090 Novosibirsk, Russia; gerasimov@catalysis.ru; 3 LISM, Moscow State Technological University Stankin, 127055 Moscow, Russia; khmelevsky@mail.ru; 4 Bauman Moscow State Technical University, 105005 Moscow, Russia; flowersova@mail.ru

**Keywords:** tin dioxide, silicon dioxide, Au clusters, hydrothermal synthesis, gas sensor, thermal stability, humid air, volatile organic compounds

## Abstract

Nanocomposites based on Au- and SiO_2_-modified SnO_2_ were studied as sensitive materials for ethanol and benzene detection in dry (RH = 1%) and humid (RH = 20%) air. Modification of SnO_2_ by amorphous SiO_2_ (13 mol.%) was effectuated by hydrothermal synthesis; modification by Au nanoparticles (1 wt.%) was carried out via impregnation by citrate-stabilized Au sol. The composition of the samples was determined by X-ray fluorescent spectroscopy and energy-dispersive X-ray spectroscopy. The microstructure was characterized by XRD, HRTEM, and low-temperature nitrogen adsorption. The surface groups were investigated by XPS, TPR-H_2_, and FTIR spectroscopy. DRIFT spectroscopy was performed to investigate the interaction between ethanol and the surface of the synthesized materials. Studies of the sensor properties have shown that in all cases the most sensitive is the SnO_2_/SiO_2_-Au nanocomposite. This material retains high sensitivity even in a humid atmosphere. The obtained results are discussed in terms of the synergistic effect of two modifiers (Au and SiO_2_) in the formation of sensor properties of SnO_2_/SiO_2_–Au nanocomposites.

## 1. Introduction

Wide-band semiconductor metal oxides have proven to be excellent sensitive materials for gas sensors. Bulk doping of these oxides or modification of their surface by various catalytic clusters can increase their sensitivity and selectivity in detecting most toxic gases [1]. In addition to high sensitivity and selectivity, the most important characteristic of a sensor material is the stability of its properties. When working with sensors based on semiconductor metal oxides, a drift in resistance values can be observed over time. The main reason for this effect is an increase in the size of the nanoparticles of a semiconductor material at operating temperatures [2,3]. As was briefly summarized in References [4,5] the introduction of SiO_2_ into the SnO_2_ semiconductor matrix is one of the promising approaches for creating sensor materials with a high stability of microstructure parameters under conditions of semiconductor gas sensors’ operation. It was found [4,5] that the modification of tin dioxide with amorphous SiO_2_, on the one hand, prevents the growth of SnO_2_ nanoparticles during high-temperature post-synthetic processing that ensures the long-term stability of sensor characteristics at high temperatures even in humid air. The temperature stability of SnO_2_/SiO_2_ nanocomposites allows it to be used for detecting gases that require a high operating temperature (300–400 °C or higher) [6,7,8]: methane, hydrogen, volatile organic compounds (VOCs), which include ethanol, benzene, toluene, and others. VOCs have a different toxicity. For example, poisoning with ethanol vapor (the threshold limit value, TLV is 522 ppm [9]) leads to difficulty breathing, nausea, headache and dizziness [10]. Mixtures of air with ethanol in a concentration of 2.6–18.9 vol% are explosive [9]. Benzene (TLV is 4.6 ppm in working zone and 1.6 ppb in residential area), which is a carcinogen, has a significantly higher toxicity. Benzene vapors at a concentration of 0.8–8.6 vol% are explosive. Chronic poisoning leads to damage to the liver and central nervous system [9]. Thus, it is extremely important to accurately and quickly detect VOCs in the air of residential and work areas. Based on the data presented in the reviews [6,8,11,12,13], the optimal detection temperatures for ethanol and benzene using metal oxide based sensors are 250–300 °C and 300–400 °C, respectively. This indicates that it is necessary to use materials that retain a high specific surface area at very high temperatures to detect these gases. This requirement is fully met by SnO_2_/SiO_2_ nanocomposites.

Gold clusters deposited on metal oxides exhibit high catalytic activity in the reactions of partial hydrogenation, synthesis of hydrogen peroxide, oxidation of CO, hydrocarbons, and also volatile organic compounds [14,15,16,17,18,19,20]. The activity of Au-based catalysts depends on many factors, including deposition methods on the oxide surface [14,21], the chemical nature of the support [22,23], the concentration [24,25,26,27] and the size [28,29] of Au nanoparticles. A summary of the literature data about the sensor properties of Au-modified SnO_2_ sensor materials towards VOCs ethanol and benzene is presented in Table 1.

This work is devoted to the study of the sensor properties of the thermally stable nanocrystalline SnO_2_/SiO_2_ composite to ethanol and benzene. To increase the sensitivity, the surface of SnO_2_/SiO_2_ nanocomposite was modified with Au nanoparticles. Based on the data from [24,25,26,27] for a combination of high catalytic activity and optimal resistance values for sensor measurements, the selected Au concentration was 1 wt.%. The most important result is that Au-modified SnO_2_/SiO_2_ retains a high sensitivity to ethanol and benzene in a humid atmosphere. Based on the DRIFTS investigations, this fact is discussed in terms of the synergistic effect of two modifiers (Au and SiO_2_) in the formation of sensor properties of the SnO_2_/SiO_2_-Au nanocomposite.

## 2. Materials and Methods

### 2.1. Materials Synthesis

**Scheme 1:**
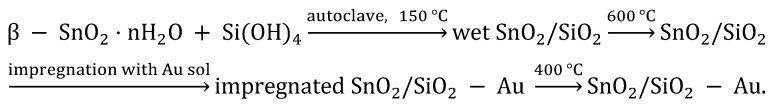
SnO_2_/SiO_2_-Au nanocomposite synthesis scheme.

It was found [4,5] that the SnO_2_/SiO_2_ nanocomposite with the composition ([Si]/([Sn] + [Si]) = 13 mol.% has optimal characteristics: a high specific surface area retained during high-temperature annealing (600 °C, 24 h); base line resistance suitable for sensor measurements; minimum sensitivity to the humidity change in the RH range from 4% to 65% over the whole range of operating temperatures. Therefore, this material has been selected as the matrix for the modification with Au nanoparticles. The synthesis of the SnO_2_/SiO_2_ nanocomposite ([Si]/([Sn] + [Si]) = 13 mol.%) was performed by hydrothermal method at 150 °C for 24 h using a xerogel of β- stannic acid SnO_2_·H_2_O and sol Si(OH)_4_. β- Stannic acid xerogel was obtained by ammonia hydrolysis of SnCl_4_·5H_2_O (98%, Sigma-Aldrich, St. Louis, MO, USA) with subsequent heat treatment at 50 °C for 24 h. Sol Si(OH)_4_ was obtained by hydrolysis of tetraethoxysilane (TEOS) (98%, Sigma-Aldrich, St. Louis, MO, USA). The synthesis method is described in detail in [4]. Post-synthetic annealing of the samples was performed at 600 °C for 24 h. Modification with Au (1 wt.%) was performed by mixing annealed SnO_2_/SiO_2_ powder and the sol of Au nanoparticles stabilized with sodium citrate, with the slow addition of a 1 M NaOH solution (>98%, Lahema, Prague, Czech Republic) to pH = 4.5–5.5. The sol of Au nanoparticles (1 mg/mL) was obtained by boiling a HAuCl_4_ solution (98%, Sigma-Aldrich, St. Louis, MO, USA) in distilled water with a solution of sodium citrate Na_3_C_6_H_5_O_7_ (>99%, Sigma-Aldrich, St. Louis, MO, USA). This method allows the obtaining of Au nanoparticles of a predetermined size [38]. Postmodification annealing was performed at 400 °C for 24 h. The sequence of synthesis stages can be described as Scheme 1.

**Scheme 2:**
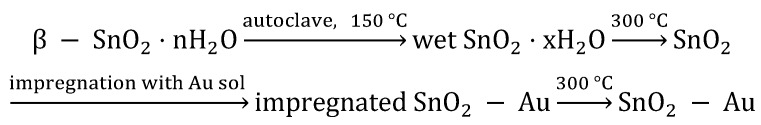
SnO_2_-Au synthesis scheme.

To determine the influence of SiO_2_ and Au on the sensor properties of tin dioxide, we also obtained SnO_2_ and SnO_2_-Au samples. Tin dioxide was obtained by annealing the xerogel of β-tin acid SnO_2_·H_2_O at 300 °C for 24 h. The modification with Au nanoparticles was carried out similarly to the preparation of SnO_2_/SiO_2_-Au nanocomposite, except that the postmodification annealing temperature in this case did not exceed 300 °C. The sequence of synthesis stages can be described as Scheme 2.

### 2.2. Materials Characterization

The Au concentration in SnO_2_-Au and SnO_2_/SiO_2_-Au samples was determined by X-ray fluorescence spectroscopy on an M1 MISTRAL instrument (Bruker, Berlin, Germany). The silicon content in SnO_2_/SiO_2_ and SnO_2_/SiO_2_-Au samples was determined by energy-dispersive X-ray spectroscopy (EDX) on a Carl Zeiss NVision 40 instrument (Carl Zeiss, Oberkochen, Germany).

The phase composition of sensitive materials was analyzed by X-ray diffraction on a DRON-4 diffractometer using monochromatic CuKα radiation (λ = 1.5406 Å) in the range of 2θ = 5–60° with a 0.1° increment. The crystallite size (d_XRD_) of the SnO_2_ phase was estimated from the broadening of the most intense XRD (101) and (110) peaks using Scherrer equation.

The microstructure of the modified nanocomposites was studied by high-resolution transmission electron microscopy on a JEM-2010 instrument (JEOL, Tokyo, Japan) with an accelerating voltage of 200 kV.

The specific surface area (S_surf_) was determined by low-temperature nitrogen adsorption on ASAP 2020 and Chemisorb 2750 instruments (Micromeritics, Norcross, GA, USA), followed by analysis using the BET model.

The surface composition was studied by IR spectroscopy, X-ray photoelectron spectroscopy (XPS), and thermoprogrammed reduction with hydrogen (TPR-H_2_). The IR spectra were recorded on a Frontier FTIR spectrometer (Perkin Elmer Inc., Waltham, MA, USA) in the transmission mode in the range of 4000–400 cm^−1^ with a 1 cm^−1^ step. The powders (1 wt.%) were grinded with dried KBr (“for FTIR analysis”, Aldrich, St. Louis, MO, USA) and pressed into tablets. IR spectra in the reflection mode during in situ studying the interaction of samples with ethanol were recorded in the range 4000–1000 cm^−1^ with a step of 1 cm^−1^ using a CdSe window. The ethanol vapor stream was set by bubbling part of the air stream through the absolute C_2_H_5_OH.

The X-ray photoelectron spectra were obtained on a K-Alpha spectrometer (ThermoFisher Scientific, Waltham, MA, USA) equipped with a monochromatic Al K_α_ X-ray source (E = 1486.7 eV). The positions of the peaks in the binding energy scale were corrected using the C1s peak corresponding to the carbon contamination of the surface (285.0 eV) with an accuracy of 0.1 eV. XP-spectra were fitted by Gaussian–Lorentzian convolution functions with the simultaneous optimization of Shirley background parameters.

The oxidative surface active sites were studied by the method of thermoprogrammed reduction with hydrogen (TPR-H_2_) on the Chemisorb 2750 (Micromeritics). The pre-treatment of the samples before the measurements was carried out in oxygen flow (20 mL/min) and included heating (10 °C/min) to 200 °C, annealing at 200 °C for 30 min, and cooling down to room temperature. During the TPR-H_2_ experiment, a H_2_/Ar gas mixture (8 vol.% H_2_) was passed through a flow-through quartz test tube with a sample. The heating was carried out up to 800 °C with a rate of 10 °C/min). The amount of hydrogen consumed in a given temperature range was calculated using calibration measurements for a standard Ag_2_O sample.

### 2.3. Study of Sensor Properties

**Figure 1:**
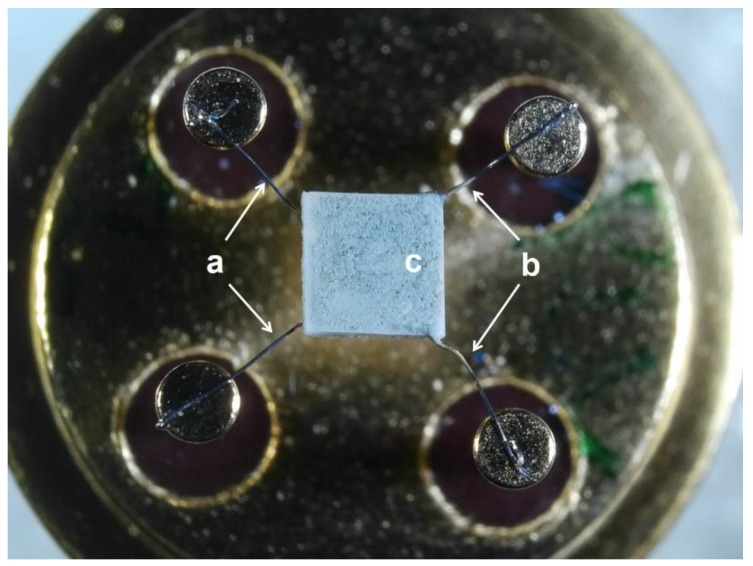
Microelectronic chip with: (**a**) platinum measuring contacts; (**b**) platinum heating contacts and (**c**) active layer of SnO_2_/SiO_2_ on the Al_2_O_3_ substrate.

For gas sensor measurements, the powders of obtained materials were mixed with α-terpeniol (90%, Kosher, SAFC, St. Louis, MO, USA) to form a paste and then deposited layer by layer on alumina substrates with platinum contacts on the top side and platinum heater on the back side (Figure 1). After each layer deposition, it was dried at a temperature of 300 °C for 3 min by applying voltage to the heating contacts. The degree of coverage was controlled through an optical microscope. In all cases, five layers were applied, which provided a total film thickness in the range of 1.0–1.2 microns. The method of applying layers of a given thickness was pre-calibrated on glass substrates. The thickness of films on glass substrates was calculated from XRF data based on a decrease in the intensity of the Ca line. Thick films thus obtained were annealed for 24 h at 300 and 400 °C in the case of SnO_2_, SnO_2_-Au and SnO_2_/SiO_2_, SnO_2_/SiO_2_-Au, respectively, by using the backside heater. At an operating temperature of 300 °C, the power consumption of the sensor element is about 0.5 W. The interaction of the obtained materials with the gas phase was studied by in situ measurement of electrical conductivity. Certified gas mixtures containing 5220 ppm ethanol and 52.5 ppm benzene in N_2_ were used as the gas sources. The flow rate of the mixture of air and the detected gas in all measurements was constant at 100 ± 0.5 mL/min. The gas concentration was set using the gas flow controllers (Bronkhorst). The period of interaction of the sensors with the gas mixture and pure air was 15 and 30 min, respectively. To control reproducibility, three cycles of measurements were performed at each temperature. A relative humidity (at 25 °C) was set and controlled by Humidifier P-2 (Cellkraft AB, Stockholm, Sweden). The response S of the sensor was calculated as S = R_air_/R_gas_, where R_gas_ is the resistance of the sample in the presence of ethanol or benzene, R_air_ is the resistance in pure air.

## 3. Results

The analysis of the obtained SnO_2_-300, SnO_2_-Au, SnO_2_/SiO_2_, SnO_2_/SiO_2_-Au samples by X-ray diffraction showed the presence of only crystalline phase SnO_2_ with the cassiterite structure (ICDD 41–1445) (Figure S1, Supplementary Information). No Au-containing phases were detected because of the low Au content. The results of the analysis of the elemental composition, microstructure parameters, and electrophysical properties of the obtained samples are shown in Table 2. The addition of SiO_2_ at the stage of hydrothermal treatment of β-tin acid allows the stabilizing of the growth of SnO_2_ nanoparticles at high-temperature annealing and samples with a high specific surface area, similar to pure SnO_2_ annealed at lower temperature 300 °C, can be obtained. The increase in the sample’s resistance in the raw R (SnO_2_-300) < R (SnO_2_-Au) < R (SnO_2_/SiO_2_) < R (SnO_2_/SiO_2_-Au) is associated with the formation of Schottky barriers at the metal/semiconductor interface and dielectric properties of silicon dioxide. When the SnO_2_/SiO_2_ surface is modified by gold, due to the difference in the electron work function of Au (5.1 eV) [39] and SnO_2_ (<4.8 eV) [40], a Schottky barrier arises at the metal/semiconductor interface. This results in an increase in the thickness of the electron-depleted layer near the surface of the SnO_2_ crystallites and, consequently, an increase in the sensor resistance.

**Figure 2:**
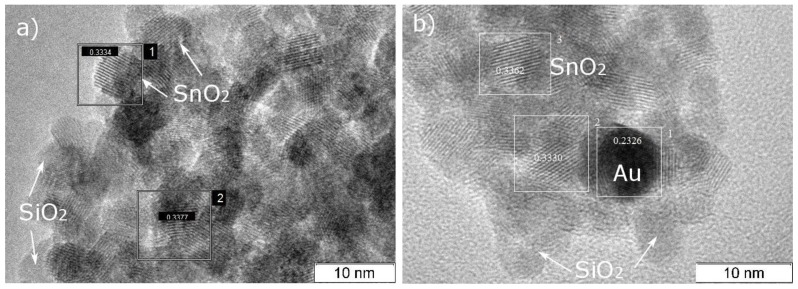
HRTEM images of samples: (**a**) SnO_2_/SiO_2_; (**b**) SnO_2_/SiO_2-_Au.

At the HRTEM image of SnO_2_/SiO_2_ (Figure 2a), the crystalline SnO_2_ phase with interplanar distances d = 0.34 nm (110) and amorphous silica particles distributed evenly on the surface of the semiconductor oxide are clearly distinguished. The cluster size of the Au modifier is one of the key factors affecting catalytic activity. According to the literature data, the optimal diameter of gold nanoparticles, which are highly active in oxidation reactions, is in the range of 1–10 nm [14,26,27,29,41]. A larger particle size leads to an insufficient degree of dispersion of the deposited modifier. According to HRTEM, the size of Au clusters on the surface of modified SnO_2_/SiO_2_ nanocomposites is 8 ± 1 nm (Figure 2b).

**Figure 3:**
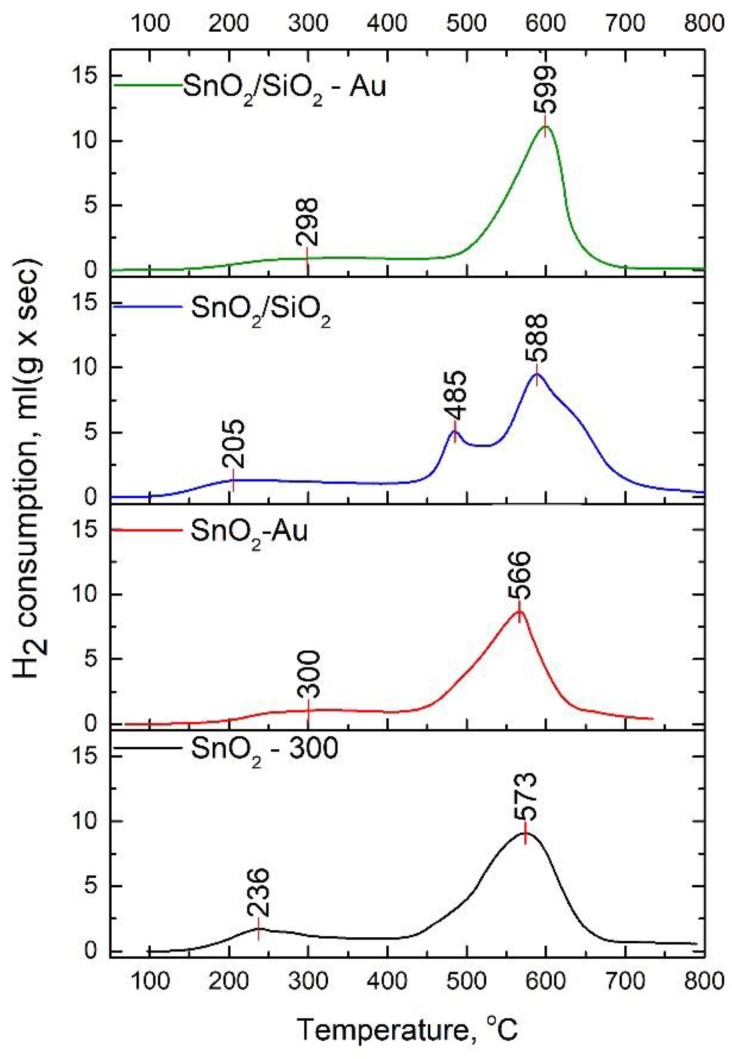
Temperature dependences of hydrogen consumption rate during reduction of SnO_2_-300, SnO_2_-Au, SnO_2_/SiO_2_ and SnO_2_/SiO_2-_Au samples.

Active sites on the surface of the samples with oxidizing properties were studied by the method of thermally programmed reduction with hydrogen (TPR-H_2_). Figure 3 shows the temperature dependences of hydrogen consumption rates for SnO_2_-300, SnO_2_-Au, SnO_2_/SiO_2_, and SnO_2_/SiO_2-_Au samples. The TPR-H_2_ profiles of all samples contain two peaks. The appearance of a peak in the low-temperature region at 170–320 °C is associated with the reduction in oxygen-containing O_2_^-^, O- and OH-groups on the surface of semiconductor materials. On TPR-H_2_ profiles of the Au-modified samples, a shift of the maximum in this peak to higher temperatures is observed by 64 °C and 93 °C in the case of SnO_2_-Au and SnO_2_/SiO_2-_Au, respectively. This effect of increasing the activation energy of the reaction of reduction of surface groups can be explained by the presence of additional reactive oxygen species and their localization in the region of the Au/SnO_2_ interface [14,42,43].

In the temperature range 450–700 °C, the consumption of hydrogen is due to a Sn^4+^ → Sn^o^ reduction. In the case of the SnO_2_/SiO_2_ sample, in a high-temperature peak, two components are distinguished. The appearance of a signal with a maximum at 485 °C is due to the partial reduction of Sn^4+^ → Sn^2+^. The maximum of the component responsible for the reduction in metal tin in SnO_2_/SiO_2_ and SnO_2_/SiO_2-_Au samples is shifted by 15 and 26 degrees, respectively, to higher temperatures, compared to the unmodified SnO_2_-300 sample, due to the presence of the SiO_2_ phase, which is resistant to reduction with hydrogen under given conditions. At the TPR-H_2_ profile of SnO_2_/SiO_2_ nanocomposite, one can distinguish an additional high-temperature shoulder at 640 °C. This may be due to the difficult reduction in tin cations linked with SiO_4_ groups. The introduction of Au clusters leads to the disappearance of this shoulder, which can indicate the weakening of the Sn-O bond on the perimeter of the SnO_2_-SiO_2_ heterocontact under the influence of the modifier. The amount of hydrogen consumed during sample reduction that was determined is shown in Table 3.

The total amount of consumed H_2_ per 1 mol of tin dioxide is close to the theoretical value (*n* = 2) determined by the reaction

SnO_2_ + 2H_2_ = Sn + 2H_2_O (1)

**Figure 4:**
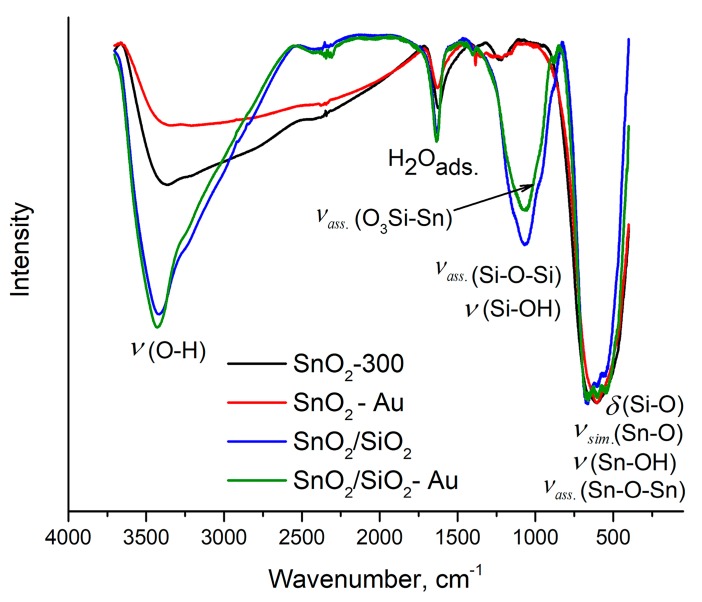
IR-spectra of SnO_2_-300, SnO_2_-Au, SnO_2_/SiO_2_ and SnO_2_/SiO_2_-Au samples.

For the reduction in oxygen-containing groups on the surface of SnO_2_/SiO_2_ and SnO_2_/SiO_2-_Au, a greater amount of hydrogen is needed than in the case of samples unmodified with silicon dioxide. This can be explained by the additional contribution of silanol Si-OH groups present on the surface of these samples. The broad absorption band with a maximum at 1100 cm^–1^ in the IR spectra of SiO_2_ containing samples is due to the superposition of antisymmetric vibrations of Si-O-Si bridge bonds and vibrations of silanol groups (1250–870 cm^–1^) (Figure 4).

**Figure 5:**
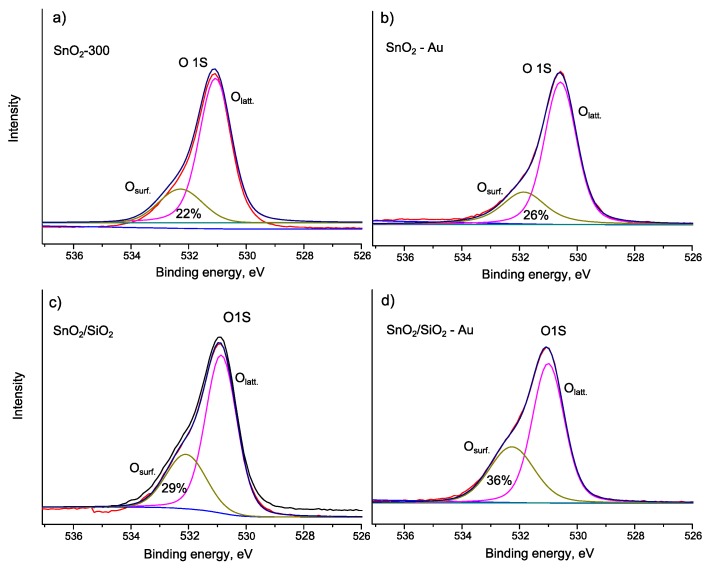
O 1s XP-spectra of: (**a**) SnO_2_-300; (**b**) SnO_2-_Au; (**c**) SnO_2_/SiO_2_ and (**d**) SnO_2_/SiO_2_-Au.

The surface composition of the obtained sensor materials was studied by the XPS method. The Sn 3d component is described by a doublet with peak maxima at 495.6 (Sn 3d_5/2_) and 487.2 (Sn3d_3/2_), which corresponds to Sn (IV) in SnO_2_ [44]. The spectra of samples containing SiO_2_ contain a signal from Si 2p with an energy of 103 eV [4]. The O 1s XP spectrum has an asymmetric form and decomposes into two peaks with energies of 531 eV (O_lat_) and 532 eV (O_surf_). The first component has a much higher intensity and corresponds to the lattice oxygen of SnO_2_. The presence of the second peak corresponds to the different forms of chemisorbed oxygen and hydroxyl groups on the surface of the obtained materials. The ratio of integrated intensities O_surf_/O_lat_ increases in the series SnO_2_-300 < SnO_2_-Au < SnO_2_/SiO_2_ < SnO_2_/SiO_2_-Au (Figure 5, Table 4), which is consistent with the data of TPR-H_2_ and IR-spectroscopy. Thus, the modification of tin dioxide by SiO_2_ and Au makes it possible to increase the concentration of surface oxygen-containing groups compared to pure SnO_2_ with the same specific surface area.

**Figure 6:**
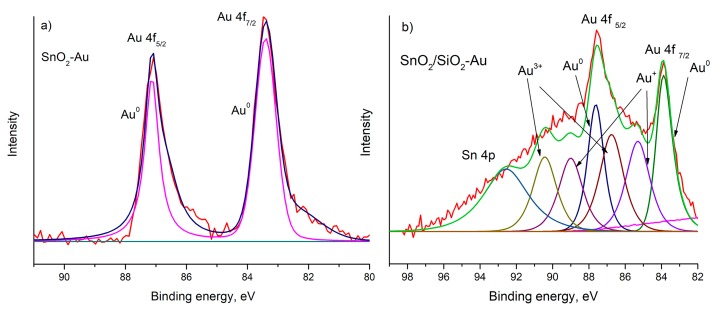
Au-component XP-spectra of: (**a**) SnO_2-_Au and (**b**) SnO_2_/SiO_2_-Au.

The signal from Au 4f_7/2_ (83.4 eV) in the SnO_2-_Au spectrum is somewhat shifted from the reference value of Au 4f_7/2_ (84.0 eV [44]). The main form of the modifier is metallic gold (Figure 6a), which is consistent with the data on the study of the charge state of gold catalysts deposited on oxide supports [45]. However, the corresponding spectral region of the SnO_2_/SiO_2_-Au sample has a more complex structure (Figure 6b), in which the components of metallic gold (83.9 eV 4f_7/2_), as well as Au^+^ (85.3 eV 4f_7/2_) and Au^3+^ cations (86.8 eV 4f_7/2_) [46] are present. The relative fractions of Au^+^ and Au^3+^ states are 11.2% and 11.4%, respectively. In addition, the region of the Au f-lines overlaps with the broad peak of Sn 4p (92.5 eV) [47]. The appearance of peaks corresponding to Au^3+^ in the SnO_2_/SiO_2_-Au spectrum can be explained by the ability of Au^3+^ (r(Au^3+^) = 0.068 nm) to integrate at the positions of coordination-unsaturated tin cations Sn_4c_ (r(Sn^4+^) = 0.069 nm) on the non-stoichiometric SnO_2_ surface. In our previous work [5], it was found that the addition of SiO_2_ in an amount of 13 mol.% leads to an increase in the concentration of paramagnetic defects Sn^3+^ by 6.8 times in comparison with pure tin dioxide. The appearance of Au_Sn_ sites leads to a change in the electron density distribution on the Sn-O bond, which is confirmed by a decrease in ΔE (Sn 3d_5/2_-O1s) for the SnO_2_/SiO_2_-Au sample by 1.1 eV (Table 4) compared to the standard value for SnO_2_ (44 eV [44]). Cabot et al. [45] found a localization of Au^δ+^ at the sites of distortion of the SnO_2_ lattice. Yinga et al. [46] described the effect of the degree of defectiveness of the carrier on the charge state of Au. An additional effect exerted by silicon dioxide is the stabilization of cationic forms of gold by hydroxyl groups, due to the formation of an Au^δ+^-OH-bond [43,48,49] whose concentration on the surface of the SnO_2_/SiO_2_-Au sample is increased compared to SnO_2_-Au due to the contribution of silanol groups. It is worth noting that it is the presence of cationic forms Au^+^ and Au^3+^ that are associated with the high catalytic activity of gold catalysts [18,29,50,51], due to the preferred adsorption of oxygen on Au^δ+^ compared to metallic gold, as well as free d-orbitals of Au^3+^.

**Figure 7:**
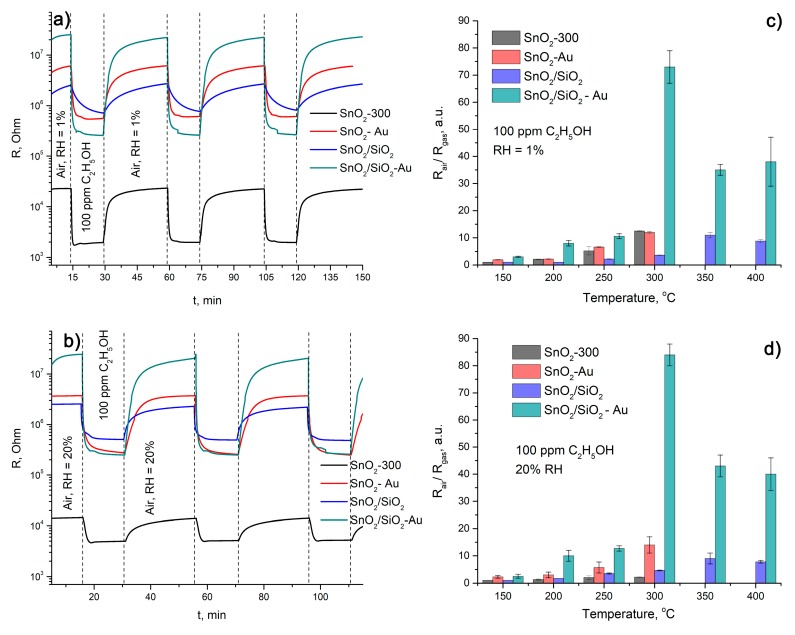
Change in the sample resistance at 300 °C in the presence of 100 ppm C_2_H_5_OH in dry air (**a**) and at RH = 20% (**b**). Temperature dependence of the sensor response in dry air (**c**) and at RH = 20% (**d**).

**Figure 8:**
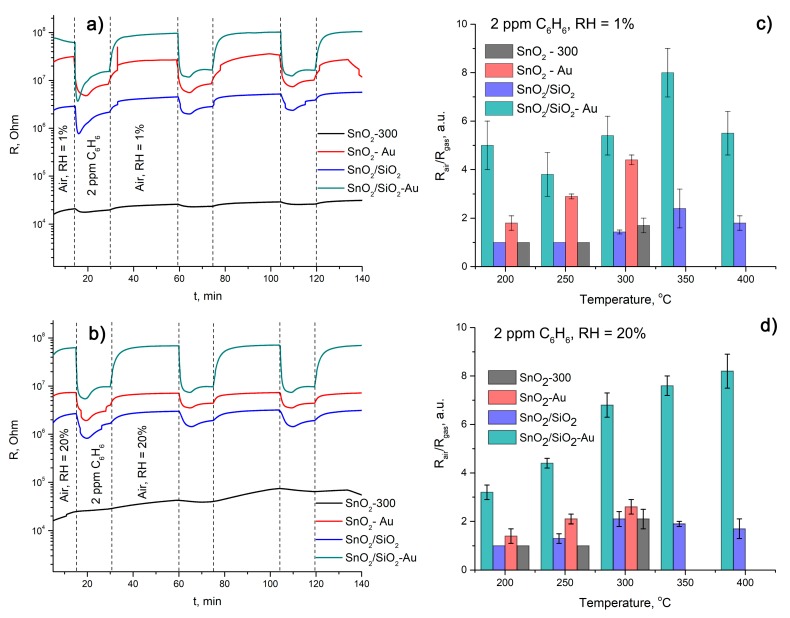
Change in the samples’ resistance at 300 °C in the presence of 2 ppm C_6_H_6_ in dry air (**a**) and at RH = 20% (**b**). Temperature dependence of the sensor response in dry air (**c**) and at RH = 20% (**d**).

The sensor properties of the obtained semiconductor materials were studied in the detection of ethanol (Figure 7) and benzene (Figure 8) in dry air (RH = 1% at 25 °C) and at a relative humidity of RH = 20% (at 25 °C) in the temperature range of 150–400 °C with increments of 50 °C. The maximum measurement temperature for sensors based on SnO_2-_300 and SnO_2-_Au did not exceed the temperature of their post-synthetic treatment, i.e., 300 °C. In the presence of ethanol vapor (Figure 7a,b), the resistance of all sensors is _reduced_. After each cycle of interaction with the gas being determined, the resistance value in air reaches the initial value. The sensor signals are reproducible (Figure 7c,d).

According to the literature data, the oxidation of ethanol on oxide surfaces proceeds in several stages. First, dissociative chemisorption of C_2_H_5_OH occurs with the formation of ethoxide by the reaction

C_2_H_5_OH_(g)_ → C_2_H_5_O_(ads)_ + H_(ads.)_. (2)

C_2_H_5_OH adsorption occurs through the oxygen atom of the hydroxyl group of the alcohol molecule on coordinatively unsaturated metal cations [52], while the hydrogen atom forms a weak bond with the surface oxygen anion. Further, depending on the conditions, ethoxide can be oxidized to acetaldehyde when reacted with chemisorbed oxygen species O- or O_2_- [17,52,53]

C_2_H_5_O_(ads)_ + O-_(ads)_ → CH_3_CHO_(ads)_ + H_2_O + e^-^ (3)

or form ethylene [52,54]:

C_2_H_5_O_(ads)_ → C_2_H_4(ads)_ + OH_(ads)_ (4)

The reactions responsible for the sensor response proceed via oxidation of organic species by O^–^ as the predominant form of chemisorbed oxygen on the SnO_2_/SiO_2_ surface at 300–400 °C [5]. The resulting acetaldehyde enters into a decarbonylation reaction with the formation of methane and carbon monoxide

CH_3_CHO_(ads)_ → CH_4(g)_ + CO_(g)_, (5)

or further oxidizes to acetic acid

(6) $$2{\mathrm{CH}}_{3}\mathrm{CHO}{}_{\left(\mathrm{ads}\right)}+5O-{}_{\left(\mathrm{ads}\right)}\;\to \;{\mathrm{CH}}_{3}\mathrm{COOH}{}_{\left(g\right)}+V_{O}^{••}.$$

The incomplete oxidation reactions (3) and (6) mainly proceed up to 220–250 °C [52,53]. At higher temperatures, ethoxide and intermediates are oxidized to carbon dioxide

CH_3_CHO_(ads)_ + 5O-_(ads)_ → 2CO_2(g)_ + 2H_2_O + 5e-, (7)

C_2_H_4(ads)_ + 6O-_(ads)_ → 2CO_2(g)_ + 2H_2_O + 6e-. (8)

The sensitivity of pure tin dioxide decreases significantly in humid air (Figure 7d), while the sensor signal of SnO_2_/SiO_2_ remains almost unchanged due to its resistance to changes in relative humidity over a wide range [4]. Modification by the Au also made it possible to neutralize the negative effects of humidity. The Au-modified SnO_2_/SiO_2_ nanocomposite demonstrates the highest sensor response to ethanol among the obtained semiconductor materials. The temperature of the maximum signal to 100 ppm C_2_H_5_OH in both dry and wet air was 300 °C (Figure 7c,d). The absolute values of the response also coincide within the error. Sensor properties of SnO_2_-300, SnO_2_-Au, SnO_2_/SiO_2_ and SnO_2_/SiO_2_-Au towards 100 ppm C_2_H_5_OH at 300 °C are summarized in Table 5. Modification by silicon dioxide prevents the increasing of sensors’ response and recovery times in humid air.

**Figure 9:**
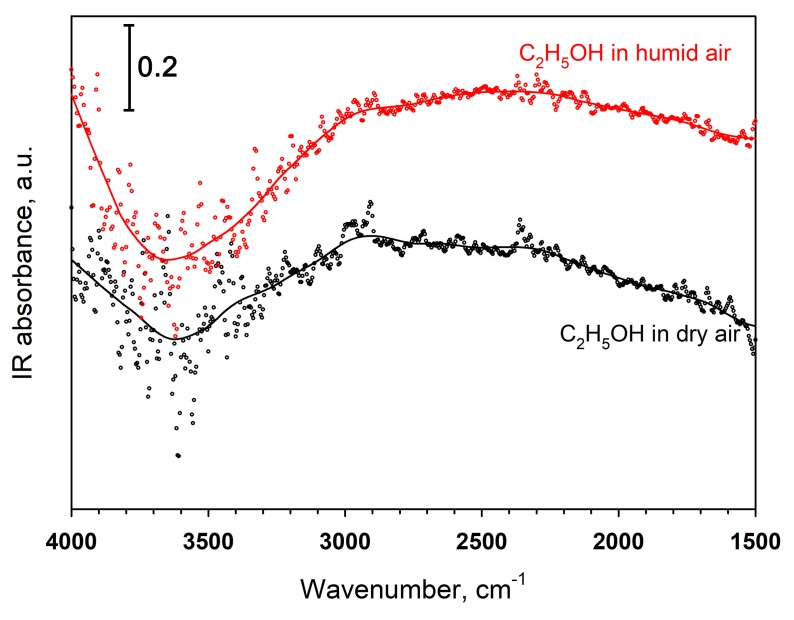
DRIFT spectra of SnO_2_/SiO_2_-Au during C_2_H_5_OH exposure at 300 °C in dry and humid air (RH = 20%).

To determine the reason for the stability of the sensor response of Au-modified SnO_2_/SiO_2_ nanocomposite with an increase in air humidity, the interaction of this material with ethanol vapor in dry and wet air was studied by DRIFT spectroscopy in operando mode. The negative change in the vibration intensities of hydroxyl groups in the range 3300–3700 cm^−1^ (Figure 9) during the interaction of the surface of the SnO_2_/SiO_2_-Au sample with C_2_H_5_OH vapor indicates their direct participation in the adsorption of ethanol molecules

C_2_H_5_OH_(ads)_ + OH_(ads)_ = C_2_H_5_O_(ads)_ + H_2_O_(g)_. (9)

Due to the high noise DRIFT IR spectra caused by an apparently more complex composition the surface of the test sample, we were not able to detect the oscillations of C-O bonds (1080–1115 cm^−1^), ν_ass._ CH_3_-, CH_2_- groups (2900–2990 cm^−1^), corresponding to adsorbed ethanol, ν_s._ CH_3_-, CH_2_- located in the region (1300–1500 cm^−1^) [53,55,56], as well as vibrations of mono- (1530–1470, 1370–1300 cm^−1^) and bidentant carbonates (1630–1590, 1270–1260 cm^−1^) [53], Sn^4+^-CO, SiO_2_-CO (2100–2200 cm^−1^), corresponding to intermediate oxidation products.

At temperatures below 250 °C, the stability of Au-modified sensor materials to increased relative humidity can be due to the involvement of water molecules adsorbed on the surface of the semiconductor oxide in the process of oxygen activation along the perimeter of the Au/SnO_2_ contact

O_2(*p*)_ + H_2_O_(surf)_ → O* + 2OH_(*p*)_, (10)

where (*p*) indicates the particles adsorbed in the region of the perimeter of the Au/SnO_2_ contact, (surf) indicates particles adsorbed on the surface of the semiconductor oxide, (*) denotes the active forms of chemisorbed oxygen [23]. DFT calculations [57,58] showed that the energy of co-adsorption of H_2_O and O_2_ on the Au (111) surface is much lower than the sum of the individual adsorption energies. The adsorption of water molecules at an increased humidity (RH = 20%) also reduces the electron work function for Au [58]. The resulting excess electrons on gold are transferred to electron-acceptor O_ads_. In sensor measurements in humid air, the effect of stabilization of the cationic forms of gold by hydroxyl groups appears. The described effects are clearly manifested in an increase in the conversion of CO oxidation reactions on Au catalysts deposited on various oxide supports at high air humidity and low temperatures (below the onset of H_2_O desorption) [23,43,59]. Another factor affecting the increase in the sensor signal to 100 ppm C_2_H_5_OH, materials modified with gold, is the possibility of the adsorption of alcohol molecules directly onto Au nanoparticles [53]. It can also be assumed that the reaction with H_ads_ [60], which was formed during the deprotonation of ethanol (Equation (2)), contributes to the formation of such a high sensor signal of the obtained semiconductor materials

H_(ads)_+ Sn_Sn_ + O-_(ads)_ ↔ Sn-OH + e-, (11)

H_(ads)_+ O_O_ ↔ (OH)_O_^+^ + e-. (12)

Sensors based on SnO_2-_300 do not show sensor sensitivity to benzene in either dry or wet air (Figure 8). A small sensor response is found for SnO_2_/SiO_2_ at high temperatures above 300 °C. Such high temperatures are caused by the stability of the benzene molecule, the complete oxidation of which is a complex multi-electron process [7]

C_6_H_6_ + 15O- → 6CO_2_ + 3H_2_O + 15e-. (13)

The modification of semiconductor materials with Au nanoparticles made it possible to detect benzene at a concentration of 2 ppm (Figure 8c,d). The sensitivity of the SnO_2-_Au sample in the atmosphere of humid air is somewhat reduced, apparently due to the competitive adsorption of C_6_H_6_ and H_2_O molecules. As in the case of ethanol, the sensor based on SnO_2_/SiO_2_-Au has the highest sensitivity and fastest response and recovery times. The temperature of the maximum sensor signal at RH = 1% was 300 °C. With an increase in relative air humidity of up to 20%, the maximum sensor response shifts to the region of higher temperatures (400 °C). This can indicate the involvement of SnO_2_ lattice oxygen in the benzene oxidation by Mars-van Krevelen mechanism [61]. The difference in energy between the lattice and surface O^2–^ is about 20 eV, while the corresponding value for O_2(gas)_ and O_2_^-^_(ads.)_ is 1.5 eV [62]. According to XPS, the modification of SnO_2_/SiO_2_ with Au leads to a decrease in the Sn-O binding energy (Table 4). The weakening of this Sn-O bond, combined with an increase in the measurement temperature, makes it possible for lattice oxygen (in addition to oxygen chemisorbates) to participate in the process of benzene oxidation, leading to a change in the resistance of the sensitive material. This mechanism was proposed by Jiang et al. [63]. The sensor properties of SnO_2_-300, SnO_2_-Au, SnO_2_/SiO_2_ and SnO_2_/SiO_2_-Au towards 2 ppm C_6_H_6_ at 300 °C are summarized in Table 6.

Thus, the synergistic effect of two modifiers (Au and SiO_2_) in the formation of sensor properties of SnO_2_/SiO_2_-Au is that the amorphous SiO_2_ provides the formation of centers on the surface of SnO_2_ that stabilize the cationic forms of gold Au^+^ and Au^3+^. These forms on the one hand exhibit higher catalytic activity in the oxidation of organic compounds, and, on the other hand, reduce the energy of the Sn-O bond that, at a high temperature, provides the involvement of SnO_2_ lattice oxygen in the benzene oxidation by Mars-van Krevelen mechanism.

## 4. Conclusions

Studies of the sensor properties of SnO_2_ - 300, SnO_2_-Au, SnO_2_/SiO_2_, SnO_2_/SiO_2_-Au nanocrystalline materials when detecting ethanol and benzene in dry air and at RH = 20% have shown that, in all cases, the most sensitive is the SnO_2_/SiO_2_–Au nanocomposite. This material retains high sensitivity even in a humid atmosphere. The modification of SnO_2_ by amorphous SiO_2_ (13 mol.%) and Au nanoparticles (1 wt.%) allowed us to obtain a number of key properties that determine the high sensor sensitivity of SnO_2_/SiO_2_-Au:

— The concentration of active oxygen-containing groups on the surface of SnO_2_/SiO_2_-Au, directly involved in the oxidation of ethanol and benzene, is the highest among all the samples, which is confirmed by XPS and TPR-H_2_;
— The addition of silicon dioxide provides long-term stability of the sensor at high temperatures, which is especially necessary for the detection of benzene;
— The addition of SiO_2_ increases the concentration of Sn^3+^ defects in tin dioxide, which, in turn, provide the formation of cationic forms Au^+^ and Au^3+^, and silanol groups are additionally involved in their stabilization. These forms are characterized by a higher catalytic activity than Au^0^;
— Similar effective ionic radii provide the possibility of Au^3+^ substitution in Sn_4c_ positions that leads to a change in the electron density distribution on the Sn-O bond. This effect also leads to the involvement of the SnO_2_ lattice oxygen in the SnO_2_/SiO_2_-Au nanocomposite in the benzene oxidation by the Mars-van Krevelen mechanism.

## Figures and Tables

**Table 1:** Sensor properties of Au-modified SnO_2_ sensor materials towards the detection of ethanol and benzene.

Material Type	Au Modification Method	Gas	C_Gas_, ppm	T_mes._, °C	Sensor Signal	Reference
nanorods	Physical vapor deposition (PVD)	C_2_H_5_OH	50	350	98	[30]
bamboo-like nanorods	multistep alternate PVD of SnO_2_ and Au				339	
powders	deposition-precipitation	C_2_H_5_OH	200	350	158	[31]
nanoflowers	impregnation	C_2_H_5_OH	100	200	123	[32]
nanoflowers	deposition-precipitation	C_2_H_5_OH	150	340	30	[33]
nanowires	γ-ray radiolysis	C_6_H_6_	1	300	3	[34]
nanofibres	electrosputtering	C_6_H_6_	10	300	2	[35]
hollow spheres	electronic beam evaporation	C_6_H_6_	5	350	22	[36]
		C_2_H_5_OH			10	
nanofibres	UV-radiation + electrospinning	C_6_H_6_	2	400	4.5	[37]

**Table 2:** Composition, microstructure parameters and electrophysical properties of the obtained samples.

Sample	Au, wt.% ^1^	SiO_2_, mol.% ^2^	Phase Composition	d_XRD_ ± 0.5 nm	S_surf_ ± 5 m^2^/g	Resistance in Air at 150^o^C, RH = 1%, 10^6^ Ohm
SnO_2_-300	-	-	SnO_2_	3.1	94	9
SnO_2_-Au	2.44 ± 0.03	-	SnO_2_	3.0	91	60
SnO_2_/SiO_2_	-	13.0 ± 0.8	SnO_2_	2.8	105	50
SnO_2_/SiO_2_-Au	3.64 ± 0.05	13.0 ± 0.8	SnO_2_	3.3	100	300

^1^ determined by X-ray fluorescence analysis; ^2^ determined by energy dispersive X-ray spectroscopy.

**Table 3:** TPR-H_2_ measurement results.

Sample	Hydrogen Consumption, mol H_2_ per 1 mol SnO_2_		
	Total	at 25 °C–400 °C	at 400 °C–800 °C
SnO_2_-300	1.9	0.2	1.7
SnO_2_-Au	1.6	0.1	1.5
SnO_2_/SiO_2_	2.3	0.3	2.0
SnO_2_/SiO_2_-Au	2.4	0.4	2.0

**Table 4:** XPS measurement results.

Sample	O_surf_/O_lat_	Binding Energy, eV			ΔE (Sn 3d_5/2-_O1s), eV
		Sn 3d_5/2_	Au 4f_7/2_	O 1s (lat)	
**SnO_2_-300**	0.28	487.2	-	531.1	43.9
**SnO_2_-Au**	0.35	486.7	83.4	530.6	43.9
**SnO_2_/SiO_2_**	0.36	487.1	-	531.1	44.0
**SnO_2_/SiO_2-_Au**	0.56	487.2	83.9	530.1	42.9

**Table 5:** Sensor properties of SnO_2_-300, SnO_2_-Au, SnO_2_/SiO_2_ and SnO_2_/SiO_2_-Au towards 100 ppm C_2_H_5_OH at 300 °C.

Sample	Sensor Response		t, resp., s		t rec., s	
	RH = 1%	RH = 20%	RH = 1%	RH = 20%	RH = 1%	RH = 20%
**SnO_2_-300**	12.5 ± 0.1	2.2 ± 0.1	83 ± 3	133 ± 2	167 ± 6	195 ± 4
**SnO_2_-Au**	12 ± 1	14 ± 3	88 ± 2	124 ± 4	164 ± 3	178 ± 6
**SnO_2_/SiO_2_**	3.6 ± 0.1	4.6 ± 0.1	352 ± 5	119 ± 3	184 ± 7	124 ± 3
**SnO_2_/SiO_2-_Au**	73 ± 6	84 ± 4	93 ± 3	90 ± 2	159 ± 5	168 ± 3

**Table 6:** Sensor properties of SnO_2_-300, SnO_2_-Au, SnO_2_/SiO_2_ and SnO_2_/SiO_2_-Au towards 100 ppm C_6_H_6_ at 300 °C.

Sample	Sensor Response		t, resp., s		t rec., s	
	RH = 1%	RH = 20%	RH = 1%	RH = 20%	RH = 1%	RH = 20%
**SnO_2_-300**	1.4 ± 0.3	1	-	-	-	-
**SnO_2_-Au**	4.4 ± 0.1	2.6 ± 0.3	97 ± 4	84 ± 8	161 ± 3	137 ± 8
**SnO_2_/SiO_2_**	1.2 ± 0.1	2.1 ± 0.3	73 ± 7	105 ± 6	142 ± 5	129 ± 4
**SnO_2_/SiO_2_-Au**	8.2 ± 0.8	6.8 ± 0.5	62 ± 5	75 ± 4	125 ± 3	128 ± 5

## Supplementary Materials

The following are available online at https://www.mdpi.com/2079-4991/10/4/813/s1, Figure S1: XRD patterns of nanocrystalline SnO_2_-300, SnO_2_-Au, SnO_2_/SiO_2_ and SnO_2_/SiO_2_-Au.
