# Supplementary material for: Synergy Effect of Au and SiO2 Modification on SnO2 Sensor Properties in VOCs Detection in Humid Air
Source: Nanomaterials (Basel). 2020 Apr 23;10(4):813. doi: 10.3390/nano10040813 (PMC7221567; doi:10.3390/nano10040813)
Supplement: Supplementary file 1 [file nanomaterials-10-00813-s001.pdf]

Synergy Effect of Au and SiO_2 Modification on SnO_2 Sensor Properties in VOCs Detection in Humid Air

Dayana Gulevich [1], Marina Rumyantseva [1,*], Evgeny Gerasimov [2], Nikolay Khmelevsky [3], Elena Tsvetkova [4] and Alexander Gaskov [1]

[1] Chemistry Department, Moscow State University, 119991 Moscow, Russia; dayana-nsu@mail.ru (D.G.); gaskov@inorg.chem.msu.ru (A.G.)
[2] Boreskov Institute of Catalysis SB RAS, 630090 Novosibirsk, Russia; gerasimov@catalysis.ru
[3] LISM, Moscow State Technological University Stankin, 127055 Moscow, Russia; khmelevsky@mail.ru
[4] Bauman Moscow State Technical University, 105005 Moscow, Russia; flowersova@mail.ru
* Correspondence: roum@inorg.chem.msu.ru; Tel.: +7-495-939-5471

Supplementary Information

The phase composition of sensitive materials was analyzed by X-ray diffraction on a DRON-4 diffractometer using monochromatic CuKα radiation (λ = 1.5406 Å) in the range of 2θ = 5–60° with 0.1° increment.

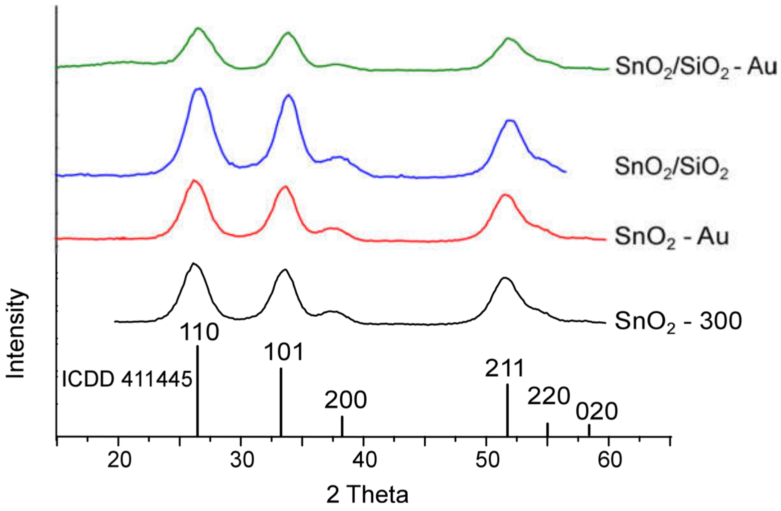

Figure S1. XRD patterns of nanocrystalline SnO_2-300, SnO_2-Au, SnO_2/SiO_2 and SnO_2/SiO_2 – Au.
